# Comparison of metaxylem vessels and pits in four sympodial bamboo species

**DOI:** 10.1038/s41598-019-47419-7

**Published:** 2019-07-26

**Authors:** Junji Luo, Caiping Lian, Rong Liu, Shuqin Zhang, Feng Yang, Benhua Fei

**Affiliations:** 1 Key Laboratory of Bamboo and Rattan Science and Technology of the State Forestry Administration, Department of Biomaterials, International Center for Bamboo and Rattan, Beijing, 100102 China; 2 School of Materials Science & Engineering, Beijing Institute of Fashion Technology, Beijing, 100029 China

**Keywords:** Natural variation in plants, Cell wall

## Abstract

The anatomical morphologies of vessel elements and pits of bamboo plants are unique, however, intensive research about vessel elements and pits in bamboo species is very scarce. The vessel elements and pits of four sympodial bamboo species were analyzed by light microscopy and environmental scanning electron microscopy (ESEM). Results show that the length and width of vessel elements were significantly different across bamboo species. The simple (main type), scalariform, and reticulate perforation plates were discovered on the end of vessel elements. The four species also displayed distinct pit forms. Characteristics of vessel elements, perforation plates, and the shape and size of pit apertures were examined separately for their potential relationship of bamboo structure and function.

## Introduction

Bamboo is one of the most important forest resources, especially in Asian countries. Bamboo grows fast and offers a good alternative to wood. The culm of bamboo can grow as tall as 36 m in 6 months, and reach its full height in one growing season^1^.

The bamboo family is very large and consists of many species. Currently, more than 1642 bamboo species have been reported worldwide, belonging to 75 genera, which compose the subfamily of *Bambusoideae*^2^. According to the structure of bamboo rhizomes, bamboo species are grouped into three different tribes: sympodial (culms aggregate closely, composing a clump), monopodial (culms are scattered) and amphipodial (culms are both aggregated and scattered)^3,4^.

The sympodial bamboos are considered to be the most primitive species of bamboo, based on the combination of their external morphological characteristics (type of rhizome, inflorescence, and culm sheath) and their internal anatomical characteristics (type of vascular bundle, mesophyll cell, and epidermal cell)^5–7^. Sympodial bamboos are distributed primarily in the southern region of China and play a vital role as materials for ornaments, building and paper pulp^4^. Due to their special biological evolutionary position and economic significance, research on the structure and function of sympodial bamboo cells are important for providing an in-depth understanding of their properties.

Previous researchers paid more attention to the relationship between the macrostructures and physical and mechanical properties of bamboo, while few detailed studies were undertaken on the anatomical features of the bamboo culm especially regarding microstructures such as the vessel elements and pits^8–11^. The structure of bamboo is considerably different from that of wood^12^. The majority of bamboo tissue in the transverse-section consists of numerous vascular bundles embedded in the parenchymatous ground tissue^7^. In the vascular bundles, various vessel elements are connected end-to-end by perforation plates to form two large metaxylem vessels. Pits refer to the regions of the cell wall in which the secondary wall is interrupted, exposing the underlying primary cell wall^13^. Vessels and pits are vital passages for the flow of xylem sap from the root to the leaves of bamboo plants^14^. The vascular bundles in bamboo are all vertically arranged, and thus the horizontal material transport depends solely on the pits. Due to the important role of pits, the ultrastructure and quantity of pits are the keys to understanding material transfer in the plants.

Over a hundred anatomical characteristics can be used to identify hardwoods and softwoods^15,16^. Likewise, metaxylem vessels and pits, along with other typical features such as the appearance of tyloses, helical thickening, the arrangement of vessel-ray pits, and the structure of perforation plates, are important characteristics used in microscopic identification of bamboo species^17–21^. However, compared to wood, there is far less relevant research in determining bamboo species using anatomical characteristics.

The aim of this study is to examine and compare key anatomical features the of metaxylem vessel elements of four sympodial bamboos, including the shape of vessel elements, the structure of perforation plates, and the ultrastructure of bordered pits on the lateral vessel wall. Morphological features of vessel elements and pits are investigated as well to determine whether they are suitable for digital visualization.

## Materials and Methods

### Bamboo samples

Four sympodial bamboo species were chosen for this study: *Neosinocalamus affinis* (Rendle) Keng, *Bambusa intermedia* Hsueh et Yi, *Bambusa multiplex* (Lour.) Raeusch. ex Schult and *Bambusa rigida* Keng et Keng f (Table 1). The *N*. *affinis*, *B*. *intermedia* and *B*. *rigida* samples were collected from bamboo forests in Ya’an and Yibin, Sichuan Province, China. The *B*. *multiplex* samples were collected from bamboo forests in Huangshan, Anhui Province, China. Three normally-growing plants per species were collected. A 3 cm long culm was cut from the middle of the sample at breast height at the internode for each plant, and the middle part of the culm wall was used for all experiments.

**Table 1:** List of bamboo samples used in the study.

Species	Sample	Year	Height(m)	DBH(cm)	Abbreviation
*Neosinocalamus affinis* (Rendle) Keng	1	4	12.80	6.37	Naf
	2	4	14.50	7.37	
	3	4	15.60	7.69	
*Bambusa intermedia* Hsueh et Yi	1	4	14.80	7.69	Bin
	2	4	16.60	6.93	
	3	4	16.50	7.67	
*Bambusa multiplex* (Lour.) Raeusch. ex Schult.	1	4	6.30	2.20	Bmu
	2	4	8.00	2.63	
	3	4	8.70	2.79	
*Bambusa rigida* Keng et Keng f.	1	4	12.90	5.09	Bri
	2	4	14.20	5.34	
	3	4	14.30	5.82	

Note: DBH refers to the diameter at breast height. All species names in this experiment have been abbreviated for simple description.

### Maceration

The samples were macerated using a previously reported method^22^. Samples of each species were split into small bamboo sticks with an approximate size of 10 mm (longitudinal) × 5 mm (radial) × 5 mm (tangential) to ensure representative selection of vessel elements. The sticks were macerated in a solution containing equal parts of glacial acetic acid (99.5%) and hydrogen peroxide (30% solution) and were subsequently heated to 60 °C for 48 h. The maceration process was ended when the color of the sticks turned white and they were easily broken into several parts. The segregated tissues were kept in a vial filled with ethanol (50%). Individual vessel elements were then selected by fine-tipped tweezers under a dissecting microscope and placed on microscope slides in order to acquire temporary specimens. The tissues were then observed by a light microscope (Leica DM LB2) and ESEM (XL30 ESEM FEG, FEI Company, US). The vessel images were photographed using a digital camera (Leica DFC300 FX).

### Observation of slabs by ESEM

Sample blocks were cut by hand with a single blade into small slabs of approximately 10 mm (longitudinal) × 5 mm (radial) × 5 mm (tangential), and then dehydrated through a graded ethanol series (30%, 50%, 70%, 85%, 100%; 5 min per solution). Each slab was then affixed to the stub with carbon paste and sputter-coated with gold for 90 sec (Leica EM SCD005). Subsequent observations were carried out using an ESEM (XL30 ESEM FEG, FEI Company, US) at an accelerating voltage of 7.9 kV.

Qualitative parameters such as perforation plates, the arrangement and type of vessel pits, and the position of pit borders and apertures, were visually evaluated by ESEM.

### Statistical analysis

Morphology of metaxylem vessels and pits of the bamboo species was evaluated. The analysis of the vessel elements focused on their length and width, and 150 vessel elements (50 vessel elements per plant) from each species were measured. Analysis of vessel pits emphasized measurements of vertical and horizontal distances of the inner and outer apertures. Over 300 vessel pits (100 vessel pits per plant) from each species were measured. To measure the dimensions of vessel pits, both outer and inner surfaces of the vessel walls were photographed. Three pictures were selected respectively from top, middle, and bottom of the vessel elements, and five pits from each picture were randomly chosen to be measured. Measurements were carried out manually with Image J software (National Institutes of Health, Bethesda, MD, USA).

The recorded data of vessel elements and pits were analyzed by SPSS. One-way ANOVA analyses were performed to test individual vessel element size and pit size of different bamboo species. During the analysis, it became evident that several attributes were not normally distributed. Because the variance was uneven, Tamhane’s T2 test was applied. All measured objects were compared in terms of significance of difference (level of significance: 5%).

## Results and Discussion

### Metaxylem vessel elements

**Figure 1:**
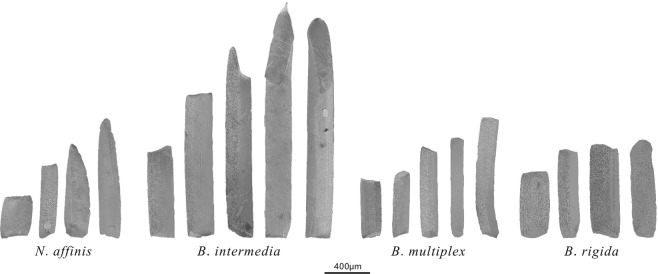
The segregated specimens are used for the comparison of vessel elements of different species.

As displayed in Fig. 1, the vessel elements of the four species obtained after the maceration varied significantly in shape and size. For each species, a total of four to five shapes were exhibited. Some of the vessels were short and broad (*N*. *affinis*) like a drum; others were slim and elongated (*B*. *intermedia*) like a stick. The vessel elements of *N*. *affinis* had tapered endings (Fig. 1), whereas those of *B*. *multiplex* and *B*. *rigida* both had smooth endings. Furthermore, it was discovered for the first time that vessel elements of bamboo (*B*. *intermedia*) possess tails, which has commonly been considered as an identifying characteristic of wood species in reports^23–25^.

**Figure 2:**
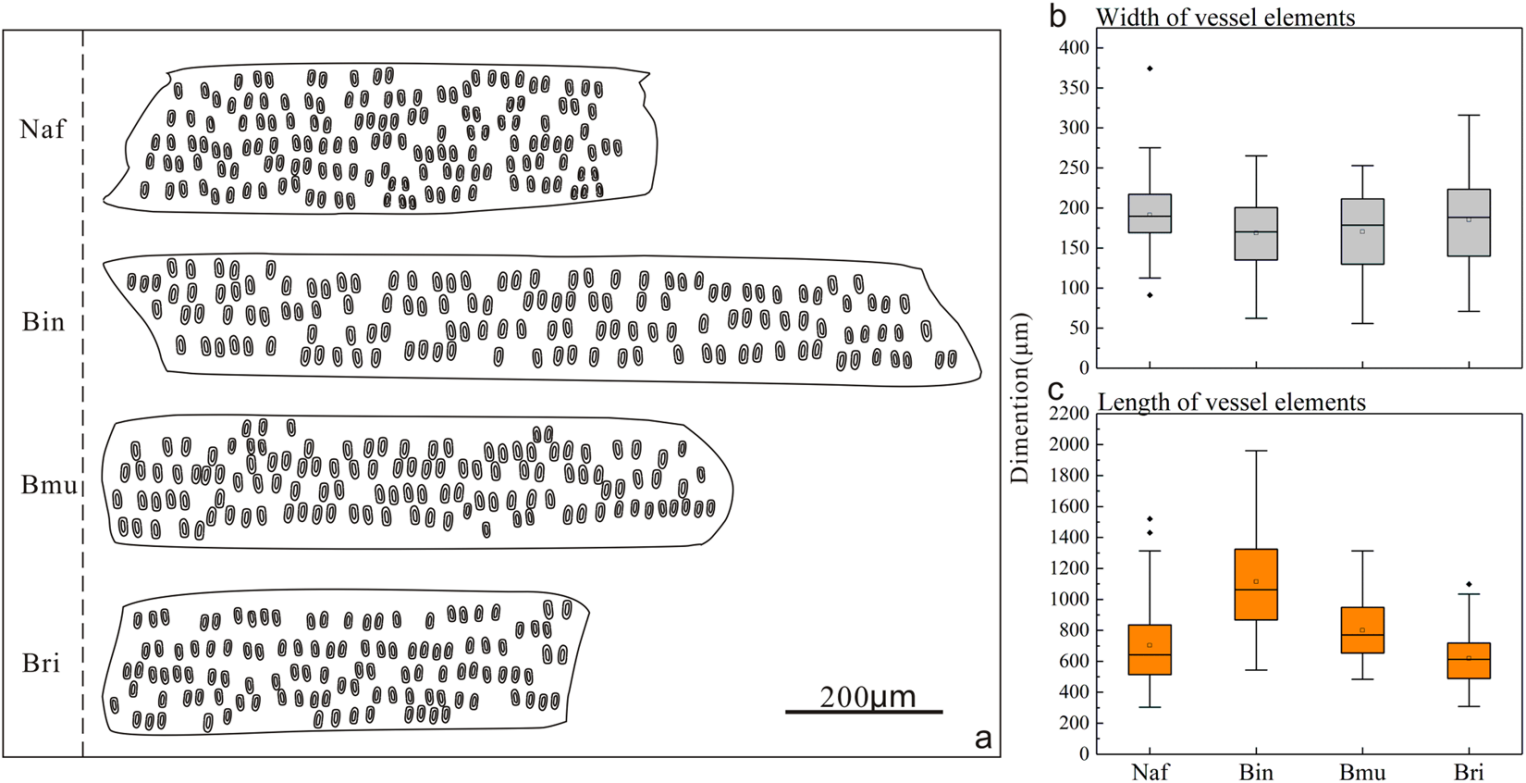
The morphologies and dimensions of the average vessel elements of *N*. *affinis* (Naf), *B*. *intermedia* (Bin), *B*. *multiplex* (Bmu), and *B*. *rigida* (Bri). (**a**) Shape of the average vessel elements based on corresponding data. (**b**) Width of the vessel elements. (**c**) Length of the vessel elements.

In order to compare the shape of the average vessel element, a sketch map was visualized and is provided in Fig. 2a. Because many diverse types were involved in the experiment, we resorted to the calculation of the average value of vessel elements for each species in order to draw a direct comparison. The results show that the average width of the vessel element had a very similar distribution in these studied species (Fig. 2b); the width ranged from 168.86 μm (*B*. *intermedia*) to 191.45 μm (*N*. *affinis*). However, the observed length of the vessel elements exhibited a wide range from 618.02 μm (*B*. *rigida*) to 1114.14 μm (*B*. *intermedia*), which was the primary reason for the different shapes of the vessel elements. After analyzing the digital and morphological features, it was noticed that vessel element diameter does not increase proportionately with length within a cell population.

The significance of the data was compared in terms of length and width of the vessel elements of the four species (Table 2). Pair-wise comparisons using the Tamhane’s T2 tests identified average vessel element length as the most significantly different characteristic between the four species. The length of *B*. *intermedia*’s vessel elements, for example, exhibited a very marked difference compared with that of *N*. *affinis* (p = 0.000 < 0.05), *B*. *multiplex* (p = 0.000 < 0.05) and *B*. *rigida* (p = 0.000 < 0.05). The same statement can be made about each one of the four sympodial bamboo species in comparison with the other three. On the contrary, the widths of the vessel elements of these four species exhibited non-significant differences. The results of the experiments on the morphological characteristics and the significance analysis indicated that although the four sympodial bamboo species had a variety of vessel element shapes, they all had relatively similar vessel element diameters. Whether similar vessel element diameters are a typical feature of sympodial bamboo species is a topic worthy of further research.

**Table 2:** Significance analysis of vessel elements and pit aperture.

Species	*B*. *intermedia*	*B*. *multiplex*	Species
			*B*. *rigida*
***N. affinis***			
Length of Vessel Elements	0.000	0.002	0.004
Width of Vessel Elements	0.000	0.000	**0**.**817**
Length of Inner Pit Apertures	0.000	0.000	0.000
Width of Inner Pit Apertures	0.000	0.000	**1**.**000**
Length of Outer Pit Apertures	0.000	0.000	0.000
Width of Outer Pit Apertures	0.000	0.000	0.000
***B. intermedia***			
Length of Vessel Elements		0.000	0.000
Width of Vessel Elements		**1**.**000**	0.035
Length of Inner Pit Apertures		**0**.**779**	0.000
Width of Inner Pit Apertures		0.000	0.000
Length of Outer Pit Apertures		0.000	**1**.**000**
Width of Outer Pit Apertures		0.000	**0**.**990**
***B. multiplex***			
Length of Vessel Elements			0.000
Width of Vessel Elements			**0**.**081**
Length of Inner Pit Apertures			0.000
Width of Inner Pit Apertures			0.000
Length of Outer Pit Apertures			0.000
Width of Outer Pit Apertures			0.000

Note: values in bold indicate no significant difference.

### The perforation plate of metaxylem vessel elements

**Figure 3:**
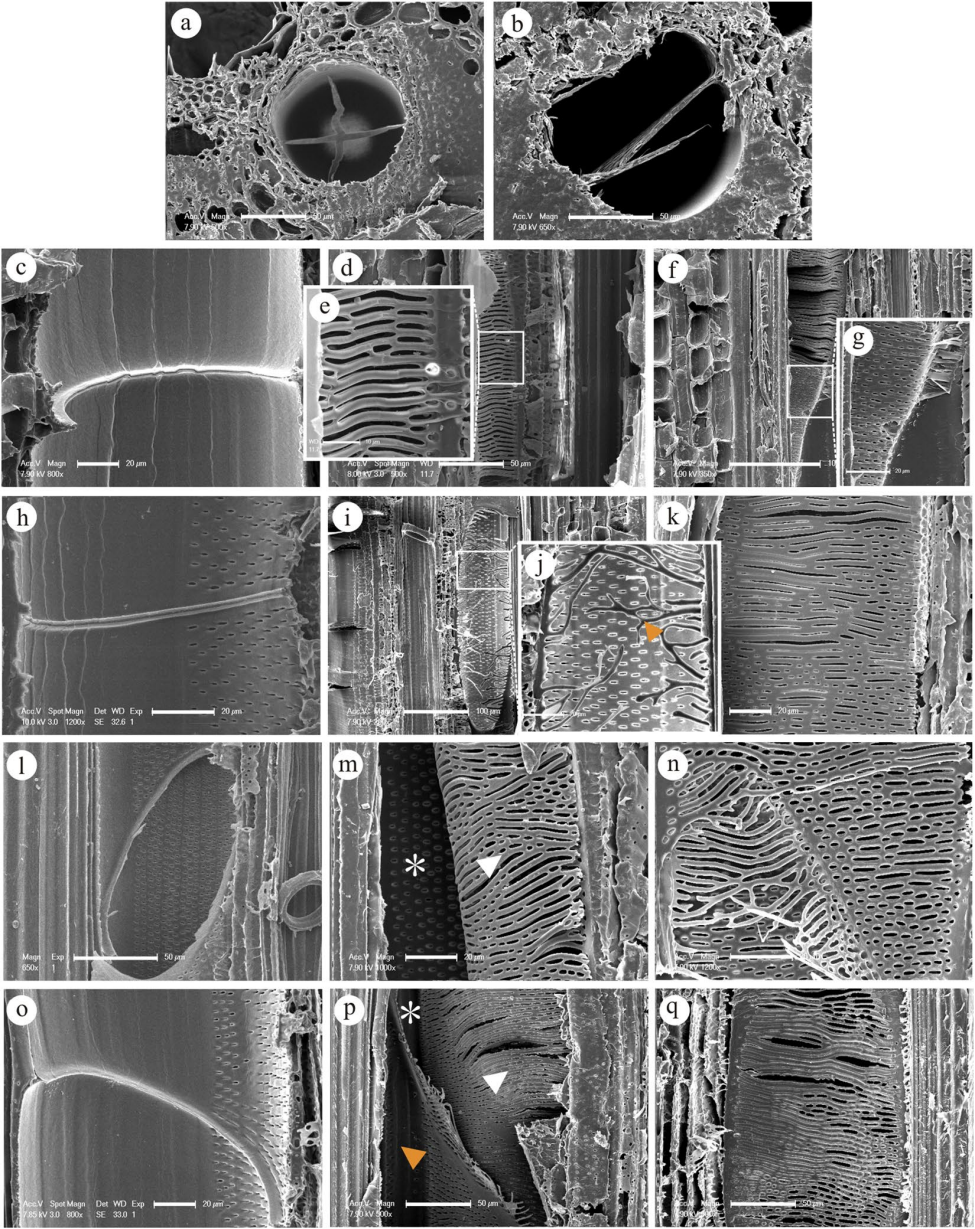
The ESEM micrographs of perforation plates of different species. (**a**,**b**) Transverse sections of simple and scalariform perforation plates (*B*. *intermedia*). (**c**–**g**) Longitudinal sections of simple and scalariform perforation plates in *N*. *affinis*. (**e**–**g**) Details of scalariform perforation plates show that the apertures appeared in two shapes: elongated and elliptical. (**h**–**k**) Longitudinal sections of simple and reticulate perforation plates in *B*. *intermedia*. (**i**–**k**) Details of scalariform perforation plates with sparse and narrow apertures. (**j**) An arrowhead indicates the incomplete bars due to sample preparation. (**l**–**n**) Longitudinal sections of simple and scalariform perforation plates in *B*. *multiplex*. (**m**) The angle was almost vertical and the scalariform perforation plates connected two vessel elements. An arrowhead indicates the scalariform perforation plates in one of the vessel element; an asterisk indicates the inner vessel wall in the other vessel element. (**o**–**q**) Longitudinal sections of simple and scalariform perforation plates in *B*. *rigida*. (**p**) The complicated scalariform perforation plates linked three vessel elements; the white arrowhead and asterisk indicate the scalariform perforation plates in two of the vessel element, respecively; an orange arrowhead indicates the inner vessel wall in the other vessel element. Bars = 50 μm (**a**), 50 μm (**b**), 20 μm (**c**), 50 μm (**d**), 10 μm (**e**), 100 μm (**f**), 20 μm (**g**), 20 μm (**h**), 100 μm (**i**), 20 μm (**j**), 20 μm (**k**), 50 μm (**l**), 20 μm (**m**), 20 μm (**n**), 20 μm (**o**), 50 μm (**p**), 50 μm (**q**).

**Figure 4:**
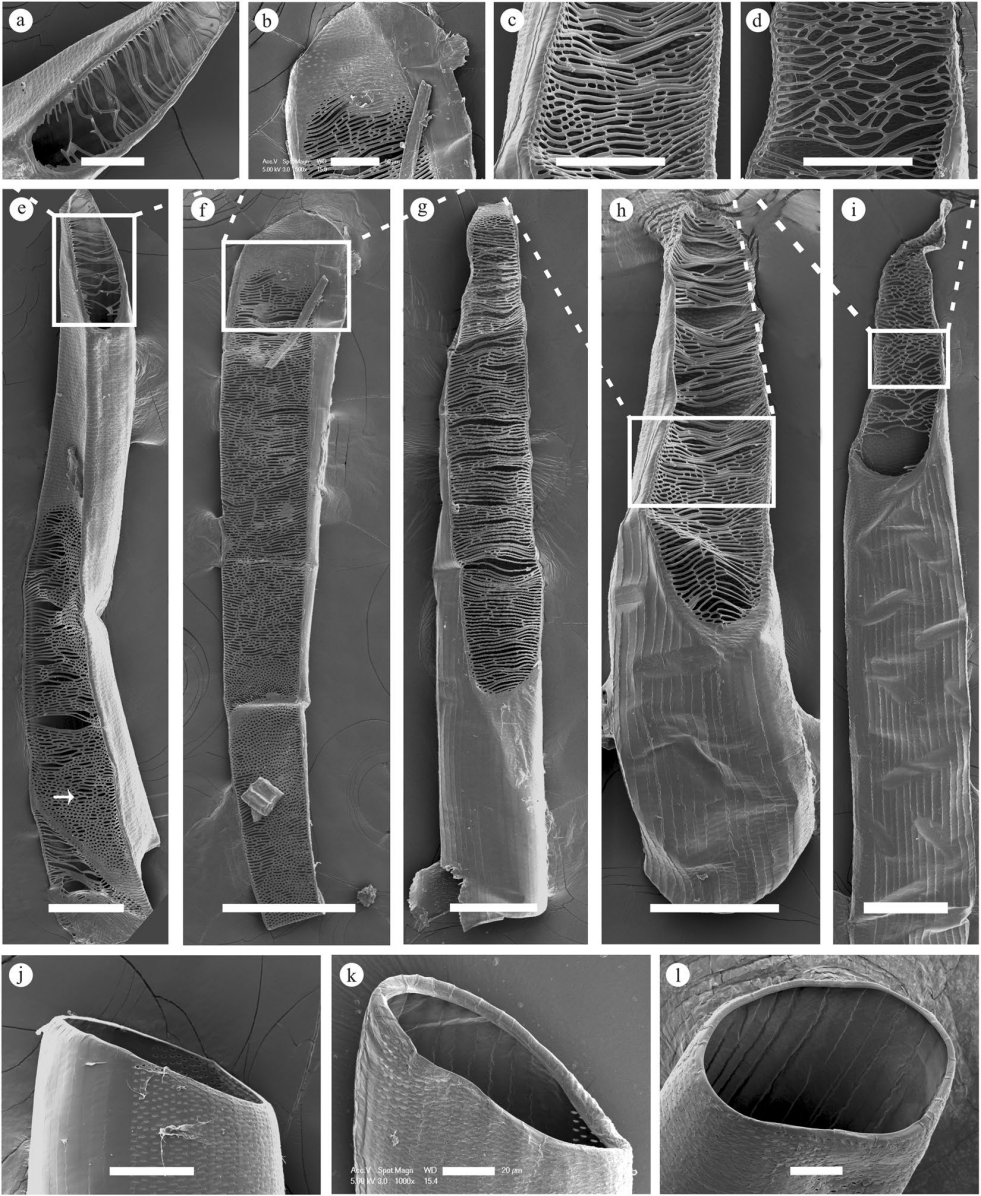
The ESEM micrographs of perforation and perforation plates of dissociated vessel elements. (**a**–**d**) Details of scalariform and reticulate perforation plates. (**e**–**i**) Independent dissociated vessel elements with different shapes of scalariform and reticulate perforation plates. (**j**–**l**) Dissociated vessel elements with simple perforation plates. (**a**) Details of scalariform perforation plates in *B*. *multiplex*. (**b**) Details of perforation plates located at the top of the vessel element in *B*. *intermedia*. (**c**) The part of the perforation plates assumes a reticulate shape in *N*. *affinis*. (**d**) Details of reticulate perforation plates in *B*. *intermedia*. (**e**) Vessel elements possess two different types of perforation plates in *B*. *multiplex*: the white box shows the scalariform perforation plates, and the arrow shows the perforation plates that are a mix of scalariform and reticulate. (**f**) Scalariform perforation plates exist across the entire side of one vessel element in *B*. *intermedia*. (**g**) Scalariform perforation plates with abundant bars in *B*. *rigida*. (**h**) Perforation plates that are a mix of scalariform and reticulate in *N*. *affinis*. (**i**) Reticulate perforation plates in *B*. *intermedia*. (**j**) Simple perforation plates with a small inclination angle in *N*. *affinis*. (**k**) Simple perforation plates with a large inclination angle in *B*. *rigida*. (**l**) Simple perforation plates assume a round shape in *B*. *intermedia*. Bars = 40 μm (**a**), 50 μm (**b**), 100 μm (**c**), 100 μm (**d**), 100 μm (**e**), 200 μm (**f**), 100 μm (**g**), 100 μm (**h**), 100 μm (**i**), 50 μm (**j**), 20 μm (**k**), 20 μm (**l**).

The term perforation plate refers to the area of the wall (originally imperforate) that is involved in the coalescence of two members of a vessel^26^. There are four types of perforation plates, namely simple perforation plates, scalariform perforation plates, reticulate perforation plates and foraminate perforation plates^20,27^. Simple, scalariform, and reticulate perforation plates were discovered on the end walls of the vessel elements of all selected bamboo species in this study. The majority of perforation plates were simple perforation plates (Fig. 3a,b). The results from the *N*. *affinis* samples were consistent with the conclusion reached by Yao^28^. Simple perforation plates were mostly horizontal or slightly inclined in angle (vertical perforation plates also existed, but only in rare cases) (Fig. 3c,h,l,o; Fig. 4j,k,l), and assumed a round or oval shape.

The extent of the variation in the scalariform perforation plates differed from species to species (Fig. 3d–g,i–k,m–p). Some scalariform perforation plates possessed elongated and parallel openings (Fig. 3d,g), while others had several short elliptical openings arranged in parallel (Fig. 3n). In addition, all the scalariform perforation plates were orientated longitudinally across the side of one vessel element so that the angle of inclination was acute and, in some cases, nearly upright (Figs 3f,i,m, 4g). Furthermore, scalariform-reticulate perforation plates were found in the long vessel element (Fig. 4c,e,h). Reticulate perforation plates were rarely discovered in *B*. *intermedia* (Fig. 4d,i). The transformation of the perforation plates from the scalariform type to the simple type was regarded as a major trend of secondary xylem evolution^29–31^. It is commonly assumed that the elimination of scalariform perforation plates results in decreased resistance to water flow^23,24,32^. Sympodial bamboos are usually distributed in southern China, where there are temperate habitats with a predominantly humid and warm climate^4^. Because bamboo can achieve rapid growth in a rather short period of time, the vessel requires a more evolved composition to accommodate such growth demand. This study found that even though simple and scalariform perforation plates both existed in all selected sympodial bamboos species, simple perforation plates appeared much more frequently than scalariform ones in all species.

### Pits in lateral walls of metaxylem vessel elements

**Figure 5:**
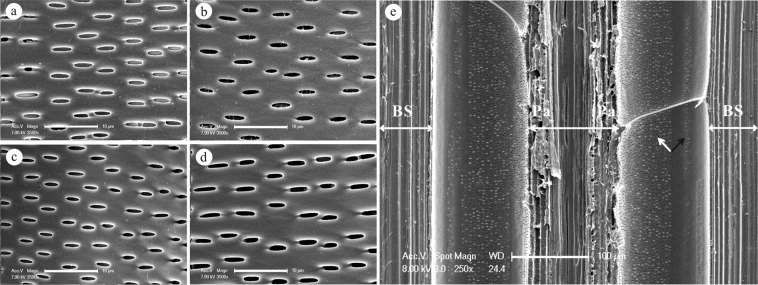
The ESEM micrographs showed pits in the inner view of the lateral metaxylem vessel elements. (**a**) The shape and arrangement of pits in *N*. *affinis*. (**b**) The shape and arrangement of pits in *B*. *intermedia*. (**c**) The shape and arrangement of pits in *B*. *multiplex*. (**d**) The shape and arrangement of pits in *B*. *rigida*. (**e**) The heterogeneity of pits adjoining different types of cells. The white arrow identifies numerous pits on the lateral wall close to the parenchyma cells, while there were fewer pits on the wall adjoining the fiber sheath in the black arrow’s direction. BS refers to bundle sheath, and Pa refers to parenchyma. Bars = 10 μm (**a**), 10 μm (**b**), 10 μm (**c**), 10 μm (**d**), 100 μm (**e**).

**Figure 6:**
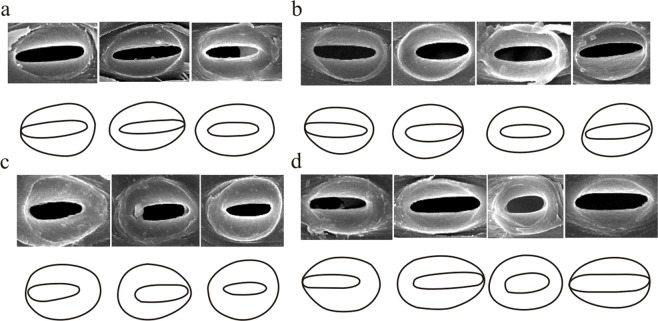
The shapes of inner and outer pits of four bamboo species. (**a**) Three types of pits were found in *N*. *affinis* (I, II, III). (**b**) Four types were found in *B*. *intermedia* (I, II, III, IV). (**c**) Three types were found in *B*. *multiplex* (I, II, III). (**d**) Tour types were found in *B*. *rigida* (I, II, III, IV).

The types and shapes of lateral cell wall pitting were also studied. In all sample species, the pits that had been observed on the lateral walls of the vessel elements were bordered pits (Table 3). This result was consistent with the findings of the same research on *Phyllostachys edulis* (Carr.) J. Houz^8^. Vessel pits were arranged in diagonal rows and the pit arrangement was mostly alternate and occasionally alternate mixed with opposite (Fig. 5a–d). The shape of the pits in the inner lateral wall was most often either flat and elliptical or with short, narrow slits (Fig. 5), whereas pits in the outer lateral wall mostly assumed a round and oval shape (Fig. 6).

**Table 3:** The morphological characteristics of pits in the lateral metaxylem vessel elements.

Species	Type	Arrangement
*N*. *affinis*	Bordered pit	Alternate
*B*. *intermedia*	Bordered pit	Alternate
*B*. *multiplex*	Bordered pit	Alternate
*B*. *rigida*	Bordered pit	Alternate

The bordered pits were divided into four types (I-IV) according to the position of inner and outer pit apertures, (Fig. 6): type I, inner pit apertures located on the right side of a border; type II, inner pit apertures located on the left side of a border; type III, inner pit apertures located between two borders; type IV, inner pit apertures located away from any border. Two species, *N*. *affinis* and *B*. *multiplex*, only had three pit types (I, II, III), while the other two *B*. *intermedia* and *B*. *rigida* had all of them.

The two large, late metaxylem vessels were in direct lateral connections with the parenchyma cells and the fiber sheath. The pit distribution in the vessel wall was obviously heterogeneous. The vessel wall adjoining the parenchyma cells displayed a large number of pits, while another part, which faced the fiber sheath, had very few to none. Sparsely pitted regions almost occupied half of the vessel circle’s circumference (Fig. 5e). A possible explanation for this phenomenon is that on one hand the parenchyma cells are part of the transport system, and the presence of abundant pits improves the efficiency of material movement. On the other hand, the fiber sheath is mostly stiff and provides mechanical support to the stem, and the presence of holes in the cell wall would significantly compromise its supporting ability^24,33,34^. This markedly contrasted pattern of pit distribution was also discovered in the metaxylem vessels of maize root^35^. It appeared to be the most efficacious way to adapt to the dual demands of material transport and mechanical support.

### The dimensions of pits

Table 4 exhibits the quantitative results, and provides the mean, minimum and maximum values of both the lengths and widths of the pit apertures of the four selected species, respectively. The pit apertures can be divided into two types: an inner pit aperture in the inner wall of the vessel elements and an outer pit aperture in the outer wall. The value differences between the minimum and maximum of both the inner and outer pit apertures were very slight in all four species (Table 4). The length of the inner pit apertures in *N*. *affinis* covered the broadest range from 1.74 to 5.62 μm. By contrast, *B*. *intermedia* had the narrowest range from 1.72 to 4.05 μm (Table 4). In general, the sizes of the outer apertures were larger than those of the inner apertures.

**Table 4:** Quantitative measurement of vessel elements and pits.

Species	Anatomical properties	Mean ± error	Min	Max
*N*. *affinis*	Length of Vessel Elements (μm)	703.37 ± 257.03	302.90	1520.09
	Width of Vessel Elements (μm)	191.45 ± 39.33	91.12	374.26
	Length of Inner Pit Apertures (μm)	3.27 ± 0.61	1.74	5.62
	Width of Inner Pit Apertures (μm)	0.83 ± 0.17	0.42	1.24
	Length of Outer Pit Apertures (μm)	4.32 ± 0.61	2.87	5.88
	Width of Outer Pit Apertures (μm)	2.77 ± 0.26	1.97	3.51
	Aspect of Inner Pit Apertures	4.07 ± 0.75	2.59	7.35
	Aspect of Outer Pit Apertures	1.57 ± 0.22	1.07	2.35
*B*. *intermedia*	Length of Vessel Elements (μm)	1114.14 ± 327.48	544.18	1960.90
	Width of Vessel Elements (μm)	168.86 ± 45.86	62.25	265.25
	Length of Inner Pit Apertures (μm)	2.83 ± 0.41	1.72	4.05
	Width of Inner Pit Apertures (μm)	0.69 ± 0.08	0.46	1.04
	Length of Outer Pit Apertures (μm)	3.96 ± 0.37	2.94	5.24
	Width of Outer Pit Apertures (μm)	2.51 ± 0.31	1.83	3.26
	Aspect of Inner Pit Apertures	4.17 ± 0.65	2.66	6.69
	Aspect of Outer Pit Apertures	1.60 ± 0.22	1.12	2.29
*B*. *multiplex*	Length of Vessel Elements (μm)	800.85 ± 194.26	483.50	1313.15
	Width of Vessel Elements (μm)	170.43 ± 47.21	55.67	253.04
	Length of Inner Pit Apertures (μm)	2.78 ± 0.61	1.60	5.30
	Width of Inner Pit Apertures (μm)	0.63 ± 0.16	0.34	1.16
	Length of Outer Pit Apertures (μm)	3.31 ± 0.47	2.21	4.96
	Width of Outer Pit Apertures (μm)	1.97 ± 0.41	1.14	2.98
	Aspect of Inner Pit Apertures	4.22 ± 0.62	2.93	6.63
	Aspect of Outer Pit Apertures	1.75 ± 0.31	1.02	3.35
*B*. *rigida*	Length of Vessel Elements (μm)	618.02 ± 158.47	307.89	1098.31
	Width of Vessel Elements (μm)	185.05 ± 54.94	70.91	316.03
	Length of Inner Pit Apertures (μm)	3.03 ± 0.55	1.81	5.54
	Width of Inner Pit Apertures (μm)	0.83 ± 0.12	0.49	1.19
	Length of Outer Pit Apertures (μm)	3.96 ± 0.55	2.52	5.61
	Width of Outer Pit Apertures (μm)	2.50 ± 0.27	1.71	3.13
	Aspect of Inner Pit Apertures	3.88 ± 0.81	2.30	7.56
	Aspect of Outer Pit Apertures	1.61 ± 0.28	1.03	2.74

**Figure 7:**
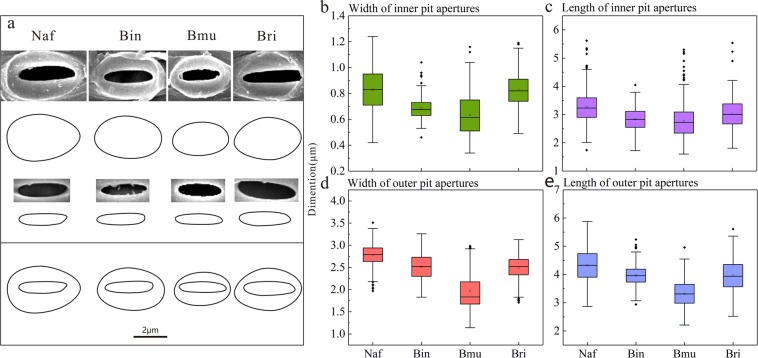
The variations of schematic and quantitative data of *N*. *affinis* (Naf), *B*. *intermedia* (Bin), *B*. *multiplex* (Bmu), and *B*. *rigida* (Bri). (**a**) Schematic of the average value of pit apertures in vessel elements. (**b**) Width of inner apertures. (**c**) Length of inner apertures. (**d**) Width of outer apertures. (**e**) Length of outer apertures.

Apart from the numerical table, the box plots below also demonstrate a sharp difference in metaxylem vessel pit measurements among the four species (Fig. 7b–e). In the box plots, the average dimensions of both the inner and outer pit apertures in *B*. *multiplex* were slightly smaller than those of the other three species. The inner pit apertures of *B*. *rigida* were the largest in size, but the outer pit apertures were not correspondingly large, and therefore the scope of the pit border was narrower than that of other species. In addition, average diameters between *N*. *affinis* and *B*. *intermedia* were very similar. These observations were also corroborated via sketch map (Fig. 7a), which depicts the mean shape of the inner and outer pit apertures of the four species.

The length and width differences of the pit apertures of these four species were compared (Table 2). The statistical evaluation according to Tamhane’s T2 test resulted in both significant and non-significant differences in the four parameters of pit apertures: 50% of the combinations were significant for all four parameters, 33.3% were significant for three of the four parameters, and only 16.7% were significant for two of the four parameters. After conducting the significance analysis and examining data from the box plots, the results indicate that the measurements of pit apertures were different in average value; however, the range of pit aperture distribution from small to large was very similar. Therefore, we concluded that sizes of the pit apertures were not the distinguishing features with which to compare the four sympodial bamboo species.

As for many bamboo species, there is a lack of data on quantitative pit characteristics. Compared with the 0.9~2.7 μm wide inner pit apertures and the 1.1~3.8 μm wide outer pit apertures in moso bamboo, the widths of the inner and outer pit apertures in the sympodial bamboo species in this study was remarkably narrower^8^. In comparison to the data on pit size in wood species, the measurement of pit apertures in sympodial bamboo species had a smaller range than both hardwoods and softwoods. The length of outer bordered pit apertures, ranges from 0.99 μm to 11μm in hardwoods^16,19,36,37^, which is greater than that of bamboo species (Table 4). The length of inner-bordered pit apertures in softwoods is reported to be in the range of 1.87 μm to 4.37 μm^38-40^, which is longer than that of sympodial bamboo species (1.60~5.62 μm) as calculated above (Table 4).

## Conclusions

In the series of vessel elements examined, our results show that the four sympodial bamboo species exhibit different shapes of vessel elements. However, most of the vessel elements are slim and elongated, which results in a similar distribution in the width of the vessel elements in the four species. In addition, variation in vessel element shape is primarily determined by differences in length. *B*. *intermedia* has the longest average vessel element (1114.14 μm), while *B*. *rigida* has the shortest (618.02 μm). Moreover, this is the first time that vessel element tails have been discovered in bamboo species (*B*. *intermedia*).

Simple, scalariform, and reticulate perforation plates exist in all selected sympodial bamboos species. The results also confirm that simple perforation plates abound in many vessel elements, whereas few vessel elements exhibit scalariform perforation plates with numerous bars. Additionally, reticulate perforation plates are rarely present in *B*. *intermedia*. Moreover, the angle of perforation plates is mostly horizontal or slightly inclined in simple perforation plates, whereas the scalariform perforation plates are more acute, or even upright, in angle.

Bordered pits exist in all sympodial bamboo species. The pit arrangement is mostly alternate, and is occasionally a mix of alternate and opposite. Four types of pits were observed, and are differentiated by the positions of the pit border and pit aperture. In order to more accurately compare the sizes of the pit apertures in these four species, we quantitatively characterized the geometry of the pits. The results found that *N*. *affinis* has the biggest mean size of the length inner and outer pit apertures (3.27 μm and 4.32 μm, respectively), while *B*. *multiplex* has the smallest (2.78 μm and 3.31 μm, respectively). Finally, we concluded that the sizes of the pit apertures were not the distinguishing features with which to compare these four sympodial bamboo species.
